# Mothers’ Vegetable Consumption Behaviors and Preferences as Factors Limiting the Possibility of Increasing Vegetable Consumption in Children in a National Sample of Polish and Romanian Respondents

**DOI:** 10.3390/nu11051078

**Published:** 2019-05-15

**Authors:** Barbara Groele, Dominika Głąbska, Krystyna Gutkowska, Dominika Guzek

**Affiliations:** 1 Department of Dietetics, Faculty of Human Nutrition and Consumer Sciences, Warsaw University of Life Sciences (SGGW-WULS), 159C Nowoursynowska Street, 02-787 Warsaw, Poland; barbara_groele@sggw.pl; 2 Department of Organization and Consumption Economics, Faculty of Human Nutrition and Consumer Sciences, Warsaw University of Life Sciences (SGGW-WULS), 159C Nowoursynowska Street, 02-787 Warsaw, Poland; krystyna_gutkowska@sggw.pl (K.G.); dominika_guzek@sggw.pl (D.G.)

**Keywords:** children, mothers, vegetable intake, consumption behaviors, choice, preferences

## Abstract

Increasing the insufficient intake of vegetables in children may be difficult, due to the influence of parents and at-home accessibility. The aim of this study was to analyze the association between self-reported vegetable consumption behaviors and preferences of mothers and the behaviors and preferences of their children, as declared by them. The nationally representative Polish (*n* = 1200) and Romanian (*n* = 1157) samples of mothers of children aged 3–10 were obtained using the random quota sampling method, and interviewed for their and their children’s general frequency of consumption and preferences of vegetables in years 2012–2014. A 24 h dietary recall of vegetable consumption was conducted for mothers and their children. Associations were observed for general number of servings consumed per day by mother–child pairs (*p* < 0.0001; *R* = 0.6522, *R* = 0.6573 for Polish and Romanian samples, respectively) and number of types indicated as preferred (*p* < 0.0001; *R* = 0.5418, *R* = 0.5433). The share of children consuming specific vegetables was 33.1–75.3% and 42.6–75.7% while their mothers also consumed, but 0.1–43.2% and 1.2–22.9% while their mothers did not. The share of children preferring specific vegetables was 16.7–74.1% and 15.2–100% when their mother shared the preference, but 1.3–46.9% and 0–38.3% when their mother did not. The mothers’ vegetable consumption behaviors and preferences may be a factor limiting the possibility of increasing vegetable consumption in their children.

## 1. Introduction

The World Health Organization (WHO) advocates regular consumption of vegetables and fruits as an important element of a child’s diet, not only in order to prevent non-communicable diet-related diseases, but also to create beneficial dietary patterns that are commonly predictive of their adolescence and adulthood patterns [1]. It is especially stated that vegetable intake patterns and preferences remain stable during childhood and adolescence [2].

Insufficient intake of vegetables and fruits is common worldwide, and the WHO has flagged it as being among the top ten determinants of global mortality [3]. At the same time, in the systematic review and meta-analysis of Touyz et al. [4], it was indicated that children more often meet the nutritional recommendations for fruits than for vegetables, so it is especially important to conduct interventions targeted at vegetables in order to increase their intake.

The ‘5-a-day’ campaign has been carried out in a number of countries in order to increase the vegetable and fruit intake of children and adults, however, both the quantity and the variety consumed are still not adequate [5]. Also, the Cochrane systematic review by Hodder et al. [6] indicated that some interventions may increase the intake of vegetables and fruits by children. However, the observed evidence was stated to be low-quality and the observed increase was stated to be minor, so future research is required [6]. Based on the analysis of consumption trends in 33 countries, it has been observed that the intake of vegetables and fruits is increasing in many countries [7]. However, the trend is not stable, as the Health Survey for England indicated an important decrease in the frequency of meeting the ‘5-a-day’ recommendation for children, from 20% to 17% between 2011 and 2013, even though an increase had been noted earlier [8]. 

Increasing the intake of vegetables in children may be hindered by a number of barriers associated with both internal and external factors [9,10] associated with sensory attributes, perception, preferences, knowledge, price, convenience, availability and accessibility, and parental, peer, and media influence. One of the most important barriers is low accessibility, which may result from seasonality [11], place of residence [12], and relatively high prices compared to other food products [13]. The other group of barriers results from preferences associated with the sensory attributes of vegetables [14], parental food consumption patterns [15], and food neophobia [16]. 

In general, a child is dependent on a diet that is prepared at home, being an element of the family environment and under general parental influence, until other influencing factors, such as peers and media, become more prominent [17]. As a result, the diets of children and parents are similar, as was observed in the systematic review and meta-analysis of Yee et al. [18], who concluded that a number of consumption behaviors of parents and their children were correlated.

For a number of the indicated barriers, the influence of parents and at-home accessibility may be crucial to reducing the children’s intake of vegetables. Such reduction of intake may be observed in spite of the fact that, in general, parents know that intake of vegetables is important for their children and believe that it will influence their health and vitality [19]. It has already been observed that in order to increase the intake of fruits by children, it is necessary to influence the fruit consumption preferences and behaviors of their mothers [20], but this has not been analyzed for vegetables so far. Taking this into account, the aim of the present study was to analyze the association between self-reported vegetable consumption behaviors and preferences of mothers and the vegetable consumption behaviors and preferences of their children, as reported by them in national samples of Polish and Romanian respondents.

## 2. Materials and Methods

### 2.1. Ethical Statement

The study was conducted on a national sample of Polish and Romanian respondents according to the guidelines laid down in the Declaration of Helsinki, and all procedures involving human subjects were approved by the Ethics Committee of the Faculty of Human Nutrition and Consumer Sciences of the Warsaw University of Life Sciences. All participants provided informed consent.

### 2.2. Studied Sample

The study was conducted on Polish and Romanian subjects, who were recruited using the same procedure and inclusion/exclusion criteria as previously described [20]. The study itself was conducted in Poland and Romania according to the same methodology, with identical questions being asked in the respondent’s native language. The data gathering was financed by the National Polish Promotion Fund for Fruits and Vegetables Consumption and Polish Association of Juices Producers within funds of the 5xVFJ (5 Portions of Vegetables, Fruit or Juice) national campaign, as an element of policy development in order to obtain the aim of increasing vegetables and fruits consumption in children.

One thousand and two hundred Polish and 1200 Romanian mothers of children aged 3–10 were planned to be recruited as respondents. The random quota sampling procedure was applied and informed consent was obtained from each respondent. Due to some missing data in the questionnaires obtained in the Romanian sample, 43 recruited respondents (3.6%), who were included in interviewing but did not complete it, were excluded. Finally, 1200 representative Polish respondents and 1157 Romanian respondents were included in years 2012–2014.

Recruitment was done in cooperation with a professional international agency that assesses public opinion and perception, and the agency was responsible for carrying out the random quota sampling. The planned quotas were determined for age, education, and residence (region and size of the city) in order to obtain representative Polish and Romanian samples of mothers of children aged 3–10.

The applied inclusion criteria were as follows: women, mother of child/children aged 3–10, inhabitant of Poland/Romania, aged 25–45. The applied exclusion criteria were as follows: lack of informed consent to participate, any missing data in the questionnaire.

### 2.3. Methods

The Computer-Assisted Telephone Interviewing (CATI) was done in cooperation with a professional international agency that assesses public opinion and perception, and the agency was hired as a partner responsible for data gathering, as in the previously described study [20]. Women aged 25–45 were randomly recruited while using the national database—they were invited to participate in the study during a telephone call and, if agreeing, they were verified for inclusion/exclusion criteria, as well as quota sampling being applied. The participation was compensated by a low value digital gift voucher code, according to commonly applied standards [21]. 

The assessment of vegetable consumption behaviors and preferences was conducted using questions that were asked about the mother’s own behaviors and preferences (self-reported) and, in separate questions, about the behaviors and preferences of her child (as reported by the mother). A mother who declared she had more than one child aged 3–10 was asked to choose one of them arbitrarily and, afterwards, to inform about vegetable consumption behaviors and preferences of only this child during the whole interview.

In spite of the fact that while parents report the intake of their children, there may be important bias associated with proxy-reporting, there is also a bias associated with self-reporting by children, as it is indicated that they are able to self-report their intake from the age of 8 [22]. As a result, it would not be effective to assess the intake self-reported by children, as in the present study, the nutritional habits of children aged 3–10 were to be assessed. In general, for younger children mothers commonly report their intake [23]. In order to not apply various methodology (proxy-reporting for younger children and self-reporting for older ones), it was decided to assess the behaviors and preferences of children as reported by mothers in all cases, as for mothers.

In addition to the questions that were planned to be analyzed, respondents were also asked additional ‘dummy questions’. They were associated with consumed vegetables, but not directly with behaviors and preferences of mothers and their children, as they were related to issues such as the place where vegetables are consumed, applied techniques of preparation of vegetables, known campaigns that promote vegetables consumption, and advantages and disadvantages of increased vegetable consumption. They were applied between main questions, in order to avoid interruptions of answers by the previous questions and answers. Respondents were also informed about the typical serving size (80 g, as defined by Food and Agriculture Organization of the United Nations (FAO) and WHO [24]) that was defined using a few examples of typical household measures for fresh and processed vegetables.

The vegetable consumption behaviors and preferences of mothers were assessed as follows:

- The general frequency of consumption of vegetables—based on the answer to the open-ended question about the number of servings of raw and processed vegetables consumed by them per day (self-reported);
- The previous day’s frequency of consumption of vegetables—based on the 24 h dietary recall of the mothers’ vegetable intake (self-reported);
- Preferred vegetables—based on the answer to the open-ended question to list the vegetables most preferred by them (self-reported);
- Consumed vegetables—based on the 24 h dietary recall of the mothers’ vegetable intake (self-reported).

The vegetable consumption behaviors and preferences of children were assessed as follows:

- The general frequency of consumption of vegetables—based on the answer to the open-ended question about the number of servings of raw and processed vegetables consumed per day by their children (reported by the mothers);
- The previous day’s frequency of consumption of vegetables—based on the 24 h dietary recall of the vegetable intake of the children (reported by the mothers);
- Preferred vegetables—based on the answer to the open-ended question to list the vegetables most preferred by their children (reported by the mothers);
- Consumed vegetables—based on the 24 h dietary recall of vegetable intake by children (reported by the mothers).

During the interview, the respondents were instructed to exclude potatoes and dry pulses from the declared number of servings of consumed vegetables. In the case of the 24 h dietary recall of vegetable intake and the list of most preferred vegetables, potatoes, dry pulses, and corn were excluded during analysis if they had been included. 

### 2.4. Statistical Analysis

The normality of the distribution was verified using the Kolmogorov–Smirnov test and, afterwards, an analysis of correlation was carried out using Spearman’s rank correlation coefficient due to the non-parametric distribution. The shares of the groups were compared using the chi^2^ test and afterwards the obtained results were controlled for the false discovery results (FDR) using the Benjamini–Hochberg procedure.

*P* ≤ 0.05 was considered significant. Statistical analysis was conducted using the following software packages: Statgraphics Plus for Windows 5.1 (Statgraphics Technologies Inc., The Plains, VA, USA), Statistica software version 8.0 (StatSoft Inc., Tulsa, OK, USA), and the Benjamini–Hochberg procedure spreadsheet by McDonalds [25].

## 3. Results

### 3.1. Analysis of the Association between the Quantity of Vegetables Consumed and Preferred by Mothers and Their Children

An analysis of the correlation between the daily frequency of vegetable consumption of the mothers and their children in nationally representative samples of Polish and Romanian mother–child pairs is presented in Table 1. 

The median for the open-ended question about general number of servings of vegetables consumed by mothers in both Polish and Romanian samples was two servings a day, and it ranged from not consuming at all to five servings a day. Similarly, for their children it was also two servings a day, and it ranged from not consuming at all (for the Polish sample) or consuming less than once a week (for the Romanian sample) to five servings a day. For both the Polish and Romanian samples, a significant correlation was observed between the number of servings consumed in general by the mothers and their children (*p* < 0.0001; *R* = 0.6522 for the Polish sample, *R* = 0.6573 for the Romanian sample).

In order to verify the association, the number of servings of vegetables consumed the previous day was analyzed, based on a 24 h dietary recall of vegetable consumption. The median for the number of servings of vegetables consumed the previous day for both mothers and their children in both Polish and Romanian samples was four servings a day, and it varied from not consuming at all to five servings a day. For both Polish and Romanian samples, a significant correlation was observed between the number of servings consumed the previous day by the mothers and their children (*p* < 0.0001; *R* = 0.4172 for the Polish sample, *R* = 0.3897 for the Romanian sample).

An analysis of the correlation between the number of types of vegetables indicated as consumed and preferred by mothers and their children in nationally representative samples of Polish and Romanian mother–child pairs is presented in Table 2. 

The median for the general number of types of vegetables indicated as consumed by the mothers in both Polish and Romanian samples was two, and it varied from a lack of types consumed to nine types. Similarly, for the children it was also two types, and it varied from a lack of types consumed to seven types (for the Polish sample) or nine types (for the Romanian sample). For both the Polish and Romanian samples, a significant correlation was observed between the number of types of vegetables indicated as consumed by mothers and their children (*p* < 0.0001; *R* = 0.5418 for the Polish sample, *R* = 0.5433 for the Romanian sample).

The median for the general number of types of vegetables indicated as preferred for both mothers and children in the Polish sample was three, and it ranged from a lack of types preferred to 19 types (for mothers) or 17 types (for children). The median for the general number of types of vegetables indicated as preferred for both mothers and children in the Romanian sample was two, and it ranged from a lack of types preferred to 17 types (for mothers) or 15 types (for children). For both the Polish and Romanian samples, a significant correlation was observed between the number of types of vegetables indicated as preferred by mothers and children (*p* < 0.0001; *R* = 0.2772 for the Polish sample, R = 0.3878 for the Romanian sample).

### 3.2. Analysis of the Association between the Variety of Vegetables Consumed and Preferred by Mothers and Their Children

An analysis of the association between the vegetable consumption behaviors self-reported by mothers and reported by them for their children in a nationally representative sample of Polish mother–child pairs is presented in Table 3. For all the vegetables that were declared by the mothers as consumed, there was a statistically significant association (*p* < 0.0001 for chi^2^ test; *p* < 0.0001 after controlling for the FDR using a Benjamini–Hochberg procedure)—types consumed by the mothers were also consumed by their children, as compared to types not consumed by the mothers. For the specific types consumed by the mothers, the consumption by the children ranged from 33.1% (for peppers) to 75.3% (for carrots), while for types not consumed by the mothers, the consumption by the children ranged from 0.1% (for eggplant) to 43.2% (for tomatoes).

An analysis of the association between the vegetable consumption behaviors self-reported by the mothers and reported by them for their children in a nationally representative sample of Romanian mother–child pairs is presented in Table 4. There was a statistically significant association (*p* < 0.0001 for chi^2^ test; *p* < 0.0001 after controlling for the FDR using a Benjamini–Hochberg procedure) for all vegetables that were declared by the mothers as consumed—types consumed by the mothers were also consumed by their children, as compared to types not consumed by the mothers. For the types consumed by the mothers, the consumption by the children ranged from 42.6% (for eggplant) to 75.7% (for carrots), while for types not consumed by the mothers, the consumption by the children ranged from 1.2% (for broccoli) to 22.9% (for carrots).

An analysis of the association between the vegetable preferences self-reported by the mothers and reported by them for their children in a nationally representative sample of Polish mother-child pairs is presented in Table 5. There was a statistically significant association (*p* < 0.002 for chi^2^ test; *p* < 0.002 after controlling for the FDR using a Benjamini-Hochberg procedure) for all vegetables that were declared by the mothers as preferred—types preferred by the mothers were also preferred by their children, as compared to types not preferred by the mothers. For specific types preferred by the mothers, children’s preferences varied from 16.7% (for eggplant) to 74.1% (for carrots), while for types not preferred by the mothers, children’s preferences ranged from 1.3% (for eggplant) to 46.9% (for carrots).

An analysis of the association between vegetable preferences self-reported by the mothers and reported by them for their children in a nationally representative sample of Romanian mother–child pairs is presented in Table 6. There was a statistically significant association (*p* < 0.0001 for chi^2^ test; *p* < 0.0001 after controlling for the FDR using a Benjamini–Hochberg procedure) for all vegetables that were declared by the mothers as preferred—types preferred by the mothers were also preferred by their children as compared to types not preferred by the mothers. For specific types preferred by the mothers, children’s preferences varied from 15.2% (for cauliflower) to 100.0% (for Chinese cabbage), while for types not preferred by the mothers, children’s preferences ranged from 0.0% (for Chinese cabbage) to 38.3% (for carrot).

## 4. Discussion

The strong associations between vegetable consumption preferences and behaviors of mothers and their children were observed both for Polish and Romanian samples. Moreover, they were observed both for assessed quantity and variety of vegetables consumed. This corresponds with the previously observed associations for fruit consumption preferences and behaviors [20], in spite of the fact that for children, fruit and vegetable preferences and exposure commonly differ [26]. As indicated by Korinek et al. [26], this is associated with children’s preference for the sweet taste and pleasant texture of fruits, and because of this, parents offer them fruits rather than vegetables, resulting in not only a higher amount, but also in a wider variety of consumed fruits as compared to vegetables. 

It is stated that repeated exposure may change the preferences, but this is more effective for fruits than vegetables [27]. Moreover, other stimuli are also more effective in increasing the intake of fruits than vegetables [28]. As a result, when comparing vegetables and fruits it may be said that, for children, fruits are not only more preferred over vegetables and a higher amount and variety is consumed, but they are also more frequently offered by parents and their intake is also easier to adopt. So, in order to assess the intake of vegetables, two domains must be analyzed—not only the consumption behaviors, but also consumption preferences, as they may be crucial for correcting nutritional behaviors.

Despite the fact that changing the preferences of vegetables may be more difficult than for fruits, it is still possible, as was proven in a number of intervention studies [29,30,31,32]. All the indicated above studies [29,30,31,32] allow us to conclude that, for children, it is possible to increase not only vegetable consumption but also preferences for the disliked ones when the exposure is applied at home. This is also confirmed by Cooke’s [33] review, which showed that both laboratory studies and interventions conducted so far for assessing the efficacy of exposure confirmed that opportunities to taste unfamiliar food products results in increased consumption and preferences. However, no national-scale studies have been conducted so far, in Poland or Romania, to analyze the association between vegetable consumption behaviors and preferences of mothers and of their children. Such observations would give broader perspective, not only to make a conclusion based on small studies of various stimuli to increase vegetable intake, but also to observe associations in real conditions.

The results of our own study indicate a significant barrier for increasing vegetable exposure at home, as the vegetable consumption behaviors of mothers determine the consumption behaviors of their children. A similar association was observed for vegetable preferences. It may be concluded that, in spite of a number of studies having proven the possibility of increasing the consumption of vegetables, our own study indicates that if mother does not like a vegetable and does not consume it, the exposure of her child to this vegetable does not exist, and this, consequently, results in lack of preference in her child.

Johnson et al. [34] classified the determinants of the size of the serving consumed by a child into child-centered (including individual preferences and general consumption behaviors of the child as well as the previous meals) and mother-centered (including their opinions about the nutritional value of products and the need to avoid wastage of money and time). Such mother-centered determinants are associated not only with child’s feeding and cooking for family members but also with her individual diet, and are observed by the child during the process of learning preferences at home [35]. However, for feeding a child there are additional factors, such as maternal feeding self-efficacy, that influence the kind and amount of products offered to the child and the child’s final eating behaviors [36].

The association between eating behaviors of mothers and their children that was observed in the conducted study is confirmed by the results of another Polish study, as a similar strong association between eating behaviors of mothers and their adolescent daughters was observed [37], and it also contributed to similar excessive body mass risk [38] and similarities in other health-related consequences [39]. This may be important, as an excessive body mass of children was commonly observed in a recent study of Polish adolescents [40]. However, a mother may also transfer her dislikes to her child and create their preferences similar to her own [41]. This may be associated with the fact that children mimic their parents in a number of behaviors, including nutritional ones [42].

Parents commonly declare that food product preferences of their children are influenced by the marketing of food, and some of them state that they make choices of food products available at home based on those preferences [43]. However, the availability of unhealthy food products at home is associated with the children’s choice of such products and the consumption of such products [44], and so if a child prefers unhealthy food products and the mother provides it, the child will consume it. At the same time, a number of parents believe that they provide such products to overcome the negative food product preferences of their children and promote a healthy diet [45]. But the question is, are they really are able to do it if their own preferences set in. A systematic review by Pearson et al. [46] indicated that vegetable and fruit consumption of children is associated not only with at home accessibility, family rules, and parental encouragement, but also with parental intake, and so parents may promote a healthy diet not by just providing it to their children but only if they also have such a diet.

The choice of food products and purchase decisions are influenced by a number of factors, including those related to health [47], nutritional knowledge [48], and the place where the product is purchased [49], but convenience and preferences may be the crucial ones [50]. This was confirmed by the results of a study by Horning et al. [51], where it was observed that the common reasons for choosing pre-packed processed meals instead of non-processed ones are preferences and time. So, even if parents declare that they want to promote healthy food decisions by their children through their own choices, other factors may interfere. 

In general, when a child asks the parent for a specific vegetable or fruit, they tend to comply with this request [52]. But for children aged 2–5 years, it was observed that food product choices based on their desire may decrease their preference for healthy food products, including vegetables, as they would rather ask about products other than vegetables [53]. As a result, parents cannot wait for their child to ask for a specific product but should provide at home accessibility. Moreover, in order to provide effective exposure, parents should not only purchase vegetables but also include them in their own diet, in order to influence their child through the role-modeling mechanism, in addition to healthy products being provided in schools and childcare institutions.

In spite of the fact that the presented study was conducted in the nationally representative samples of Polish and Romanian respondents, some limitations must be indicated. The main limitations are associated with the proxy-reporting, due to the fact that the consumption preferences and behaviors of children were declared by their mothers. Moreover, there were the different participation rates in the Polish and Romanian samples, with some missing data in the Romanian sample. It must also be indicated that the mother was able to arbitrarily choose which of her children to discuss, with no randomization. Moreover, the 24 h dietary recall of vegetable consumption turned out to be a tool that over reported the intake, so results of general declared intake rather than the previous day’s intake should be taken into account. All the indicated issues may result in some bias and must be taken into account in the further studies.

## 5. Conclusions

In Polish and Romanian representative samples of mother–child pairs, it was observed that vegetable consumption preferences and behaviors of mothers and their children were associated, both for the quantity and variety of consumed vegetables. A mother’s lack of preference for specific vegetables may cause a lack of at-home accessibility (namely, lack of exposure for their children) and a resultant lack of preference of their children. In order to increase the vegetable intake in a child’s diet, effective exposure should be provided, not only by purchasing the products and by at home accessibility (exposure), but also by including them in the diet of parents (role-modeling).

## Figures and Tables

**Table 1:** Analysis of the correlation between the daily frequency of vegetables consumption of mothers and of their children in nationally representative samples of Polish (*n* = 1200) and Romanian (*n* = 1157) mother–child pairs.

Analyzed Correlation		*p*-Value ^a^	*R*
Polish mother–child pairs (*n* = 1200)	general daily frequency	<0.0001	0.6522
	previous day frequency	<0.0001	0.4172
Romanian mother–child pairs (*n* = 1157)	general daily frequency	<0.0001	0.6573
	previous day frequency	<0.0001	0.3897

^a^ Spearman’s rank correlation coefficient.

**Table 2:** Analysis of the correlation between the number of types of vegetables indicated as consumed and preferred by mothers and by their children in nationally representative samples of Polish (*n* = 1200) and Romanian (*n* = 1157) mother–child pairs.

Analyzed Correlation		*p*-Value ^a^	*R*
Polish mother–child pairs (*n* = 1200)	number of vegetables indicated as consumed	<0.0001	0.5418
	number of vegetables indicated as preferred	<0.0001	0.2872
Romanian mother–child pairs (*n* = 1157)	number of vegetables indicated as consumed	<0.0001	0.5433
	number of vegetables indicated as preferred	<0.0001	0.3878

^a^ Spearman’s rank correlation coefficient.

**Table 3:** Analysis of the association between the vegetable consumption behaviors self-reported by the mothers and reported by them for their children in a nationally representative sample of Polish mother–child pairs (*n* = 1200).

Vegetable ^a^	Mothers Consuming the Specified Vegetable ^b^		Mothers not Consuming the Specified Vegetable ^b^		*p*-Value ^c^
	Reporting their Children as also Consuming	Reporting their Children as not Consuming	Reporting their Children as Consuming	Reporting their Children as also not Consuming	
Carrot (*n* = 592; *n* = 608)	446 (75.3%)	146 (24.7%)	141 (23.2%)	467 (76.8%)	<0.0001
Tomato (*n* = 393; *n* = 807)	231 (58.8%)	162 (41.2%)	349 (43.2%)	458 (56.8%)	<0.0001
Cucumber (*n* = 325; *n* = 875)	196 (60.3%)	129 (39.7%)	330 (37.7%)	545 (62.3%)	<0.0001
Cabbage (*n* = 167; *n* = 1033)	90 (53.9%)	77 (46.1%)	64 (6.2%)	969 (93.8%)	<0.0001
Pepper (*n* = 133; *n* = 1067)	44 (33.1%)	89 (66.9%)	32 (3.0%)	1035 (97.0%)	<0.0001
Lettuce (*n* = 120; *n* = 1080)	48 (40.0%)	72 (60.0%)	48 (4.4%)	1032 (95.6%)	<0.0001
Celery (*n* = 103; *n* = 1097)	40 (38.8%)	63 (61.2%)	25 (2.3%)	1072 (97.7%)	<0.0001
Beetroot (*n* = 103; *n* = 1097)	73 (70.9%)	30 (29.1%)	52 (4.7%)	1045 (95.3%)	<0.0001
Onion (*n* = 97; *n* = 1103)	47 (48.5%)	50 (51.5%)	34 (3.1%)	1069 (96.9%)	<0.0001
Broccoli (*n* = 88; *n* = 1112)	38 (43.2%)	50 (56.8%)	16 (1.4%)	1096 (98.6%)	<0.0001
Cauliflower (*n* = 66; *n* = 1134)	34 (51.5%)	32 (48.5%)	32 (2.8%)	1102 (97.2%)	<0.0001
Chinese cabbage (*n* = 45; *n* = 1155)	20 (44.4%)	25 (55.6%)	15 (1.3%)	1140 (98.7%)	<0.0001
Green peas (*n* = 38; *n* = 1162)	18 (47.4%)	20 (52.6%)	19 (1.6%)	1143 (98.4%)	<0.0001
Beans (*n* = 27; *n* = 1173)	11 (40.7%)	16 (59.3%)	12 (1%)	1161 (99%)	<0.0001
Zucchini (*n* = 23; *n* = 1177)	13 (56.5%)	10 (43.5%)	2 (0.2%)	1175 (99.8%)	<0.0001
Eggplant (*n* = 5; *n* = 1195)	3 (60.0%)	2 (40.0%)	1 (0.1%)	1194 (99.9%)	<0.0001

^a^ the number of mothers consuming the specific vegetable, followed by the number of mothers non-consuming the specific vegetable; ^b^ assessed based on the previous day’s vegetable consumption behaviors; ^c^ chi^2^ test.

**Table 4:** Analysis of the association between the vegetable consumption behaviors self-reported by the mothers and reported by them for their children, in a nationally representative sample of Romanian mother–child pairs (*n* = 1157).

Vegetable ^a^	Mothers Consuming the Specified Vegetable ^b^		Mothers not Consuming the Specified Vegetable ^b^		*p*-Value ^c^
	Reporting their Children as also Consuming	Reporting their Children as not Consuming	Reporting their Children as Consuming	Reporting their Children as also not Consuming	
Carrot (*n* = 568; *n* = 589)	430 (75.7%)	138 (24.3%)	135 (22.9%)	454 (77.1%)	<0.0001
Pepper (*n* = 429; *n* = 728)	254 (59.2%)	175 (40.8%)	102 (14.0%)	626 (86.0%)	<0.0001
Tomato (*n* = 406; *n* = 751)	243 (59.9%)	163 (40.1%)	93 (12.4%)	658 (87.6%)	<0.0001
Onion (*n* = 324; *n* = 833)	177 (54.6%)	147 (45.4%)	73 (8.8%)	760 (91.2%)	<0.0001
Cucumber (*n* = 200; *n* = 957)	116 (58.0%)	84 (42.0%)	81 (8.5%)	876 (91.5%)	<0.0001
Celery (*n* = 191; *n* = 966)	104 (54.5%)	87 (45.5%)	60 (6.2%)	906 (93.8%)	<0.0001
Cabbage (*n* = 103; *n* = 1054)	56 (54.4%)	47 (45.6%)	44 (4.2%)	1010 (95.8%)	<0.0001
Beans (*n* = 91; *n* = 1066)	44 (48.4%)	47 (51.6%)	26 (2.4%)	1040 (97.6%)	<0.0001
Green peas (*n* = 63; *n* = 1094)	42 (66.7%)	21 (33.3%)	27 (2.5%)	1067 (97.5%)	<0.0001
Eggplant (*n* = 61; *n* = 1096)	26 (42.6%)	35 (57.4%)	17 (1.6%)	1079 (98.4%)	<0.0001
Zucchini (*n* = 49; *n* = 1108)	23 (46.9%)	26 (53.1%)	27 (2.4%)	1081 (97.6%)	<0.0001
Lettuce (*n* = 51; *n* = 1106)	18 (35.3%)	33 (64.7%)	20 (1.8%)	1086 (98.2%)	<0.0001
Cauliflower (*n* = 35; *n* = 1122)	16 (45.7%)	19 (54.3%)	16 (1.4%)	1106 (98.6%)	<0.0001
Broccoli (*n* = 27; *n* = 1130)	17 (63.0%)	10 (37.0%)	13 (1.2%)	1117 (98.8%)	<0.0001
Beetroot (*n* = 22; *n* = 1135)	15 (68.2%)	7 (31.8%)	17 (1.5%)	1118 (98.5%)	<0.0001

^a^ the number of mothers consuming the specific vegetable followed by the number of mothers non-consuming the specific vegetable; ^b^ assessed based on the previous day’s vegetable consumption behaviors; ^c^ chi^2^ test.

**Table 5:** Analysis of the association between the vegetable preferences self-reported by the mothers and reported by them for their children in a national representative sample of Polish mother–child pairs (*n* = 1200).

Vegetable ^a^	Mothers Indicating the Specified Vegetable as the Most Preferred		Mothers not Indicating the Specified Vegetable as the Most Preferred		*p*-Value ^b^
	Reporting their Children as also Preferring	Reporting their Children as not Preferring	Reporting their Children as Preferring	Reporting their Children as also not Preferring	
Carrot (*n* = 665; *n* = 535)	493 (74.1%)	172 (25.9%)	251 (46.9%)	284 (53.1%)	<0.0001
Tomato (*n* = 576; *n* = 624)	342 (59.4%)	234 (40.6%)	222 (35.6%)	402 (64.4%)	<0.0001
Cucumber (*n* = 433; *n* = 767)	313 (72.3%)	120 (27.7%)	345 (45.0%)	422 (55.0%)	<0.0001
Broccoli (*n* = 279; *n* = 921)	97 (34.8%)	182 (65.2%)	112 (12.2%)	809 (87.8%)	<0.0001
Cauliflower (*n* = 269; *n* = 931)	123 (45.7%)	146 (54.3%)	192 (20.6%)	739 (79.4%)	<0.0001
Beetroot (*n* = 205; *n* = 995)	88 (42.9%)	117 (57.1%)	236 (23.7%)	759 (76.3%)	<0.0001
Cabbage (*n* = 191; *n* = 1009)	71 (37.2%)	120 (62.8%)	154 (15.3%)	855 (84.7%)	<0.0001
Lettuce (*n* = 181; *n* = 1010)	58 (32.0%)	123 (68%)	166 (16.3%)	853 (83.7%)	<0.0001
Pepper (*n* = 153; *n* = 1047)	56 (36.6%)	97 (63.4%)	143 (13.7%)	904 (86.3%)	<0.0001
Beans (*n* = 92; *n* = 1108)	41 (44.6%)	51 (55.4%)	133 (12.0%)	975 (88.0%)	<0.0001
Chinese cabbage (*n* = 77; *n* = 1123)	21 (27.3%)	56 (72.7%)	147 (13.1%)	976 (86.9%)	0.0010
Celery (*n* = 77; *n* = 1123)	15 (19.5%)	62 (80.5%)	48 (4.3%)	1075 (95.7%)	<0.0001
Onion (*n* = 71; *n* = 1129)	14 (19.7%)	57 (80.3%)	79 (7.0%)	1050 (93.0%)	0.0003
Zucchini (*n* = 62; *n* = 1138)	12 (19.4%)	50 (80.6%)	86 (7.6%)	1052 (92.4%)	0.0022
Green peas (*n* = 48; *n* = 1152)	21 (43.8%)	27 (56.3%)	146 (12.7%)	1006 (87.3%)	<0.0001
Eggplant (*n* = 24; *n* = 1176)	4 (16.7%)	20 (83.3%)	15 (1.3%)	1161 (98.7%)	<0.0001

^a^ the number of mothers preferring the specific vegetable followed by the number of mothers non-preferring the specific vegetable; ^b^ chi^2^ test.

**Table 6:** Analysis of the association between the vegetable preferences self-reported by the mothers and reported by them for their children in a nationally representative sample of Romanian mother–child pairs (*n* = 1157).

Vegetable ^a^	Mothers Indicating the Specified Vegetable as the Most Preferred		Mothers not Indicating the Specified Vegetable as the Most Preferred		*p*-Value ^b^
	Reporting their Children as also Preferring	Reporting their Children as not Preferring	Reporting their Children as Preferring	Reporting their Children as also not Preferring	
Tomato (*n* = 571; *n* = 586)	289 (50.6%)	282 (49.4%)	108 (18.4%)	478 (81.6%)	<0.0001
Carrot (*n* = 447; *n* = 710)	328 (73.4%)	119 (26.6%)	272 (38.3%)	438 (61.7%)	<0.0001
Pepper (*n* = 378; *n* = 779)	136 (36.0%)	242 (64.0%)	111 (14.2%)	668 (85.8%)	<0.0001
Cucumber (*n* = 341; *n* = 816)	191 (56.0%)	150 (44.0%)	184 (22.5%)	632 (77.5%)	<0.0001
Cabbage (*n* = 157; *n* = 1000)	60 (38.2%)	97 (61.8%)	81 (8.1%)	919 (91.9%)	<0.0001
Eggplant (*n* = 140; *n* = 1017)	32 (22.9%)	108 (77.1%)	34 (3.3%)	983 (96.7%)	<0.0001
Onion (*n* = 130; *n* = 1027)	30 (23.1%)	100 (76.9%)	43 (4.2%)	984 (95.8%)	<0.0001
Cauliflower (*n* = 105; *n* = 1052)	16 (15.2%)	89 (84.8%)	29 (2.8%)	1023 (97.2%)	<0.0001
Beans (*n* = 91; *n* = 1066)	24 (26.4%)	67 (73.6%)	32 (3%)	1034 (97%)	<0.0001
Lettuce (*n* = 87; *n* = 1070)	17 (19.5%)	70 (80.5%)	25 (2.3%)	1045 (97.7%)	<0.0001
Celery (*n* = 87; *n* = 1070)	14 (16.1%)	73 (83.9%)	14 (1.3%)	1056 (98.7%)	<0.0001
Green peas (*n* = 69; *n* = 1088)	24 (34.8%)	45 (65.2%)	33 (3%)	1055 (97%)	<0.0001
Zucchini (*n* = 64; *n* = 1093)	20 (31.3%)	44 (68.8%)	23 (2.1%)	1070 (97.9%)	<0.0001
Broccoli (*n* = 63; *n* = 1094)	20 (31.7%)	43 (68.3%)	14 (1.3%)	1080 (98.7%)	<0.0001
Beetroot (*n* = 35; *n* = 1122)	7 (20.0%)	28 (80.0%)	16 (1.4%)	1106 (98.6%)	<0.0001
Chinese cabbage (*n* = 1; *n* = 1156)	1 (100.0%)	0 (0.0%)	0 (0.0%)	1156 (100.0%)	<0.0001

^a^ the number of mothers preferring the specific vegetable followed by the number of mothers non-preferring the specific vegetable; ^b^ chi^2^ test.
